# Brief Report: Which Came First? Exploring Crossmodal Temporal Order Judgements and Their Relationship with Sensory Reactivity in Autism and Neurotypicals

**DOI:** 10.1007/s10803-016-2925-z

**Published:** 2016-10-04

**Authors:** Daniel Poole, Emma Gowen, Paul A. Warren, Ellen Poliakoff

**Affiliations:** Division of Neuroscience and Experimental Psychology, University of Manchester, Oxford Road, M13 9PL Manchester, UK

**Keywords:** Autism, Crossmodal temporal order judgements, Sensory reactivity, Crossmodal bias, Temporal acuity, Multisensory

## Abstract

Previous studies have indicated that visual-auditory temporal acuity is reduced in children with autism spectrum conditions (ASC) in comparison to neurotypicals. In the present study we investigated temporal acuity for all possible bimodal pairings of visual, tactile and auditory information in adults with ASC (n = 18) and a matched control group (n = 18). No group differences in temporal acuity for crossmodal stimuli were observed, suggesting that this may be typical in adults with ASC. However, visual-tactile temporal acuity and bias towards vision when presented with visual-auditory information were both predictors of self-reported sensory reactivity. This suggests that reduced multisensory temporal acuity and/or attention towards vision may contribute to atypical sensory reactivity.

## Introduction

Atypical reactivity across multiple sensory modalities (e.g. vision, touch, hearing) is widely reported in autism spectrum condition (ASC; O’Neill and Jones 1997). This includes hyper and hypo sensitivity to sensory information, and sensory seeking behaviours (Lane et al. 2010). Although differences in sensory reactivity have been observed since the early descriptions of ASC, the aetiology, and exact nature, of these differences remains unknown. Recently, it has been suggested that an atypical interaction *between* information from the different senses may account for these differences (Iarocci and McDonald 2006), and in particular that temporal acuity (the ability to separate stimuli in time) between the senses may be reduced (see Stevenson et al. 2015 for a review).

**Fig. 1:**
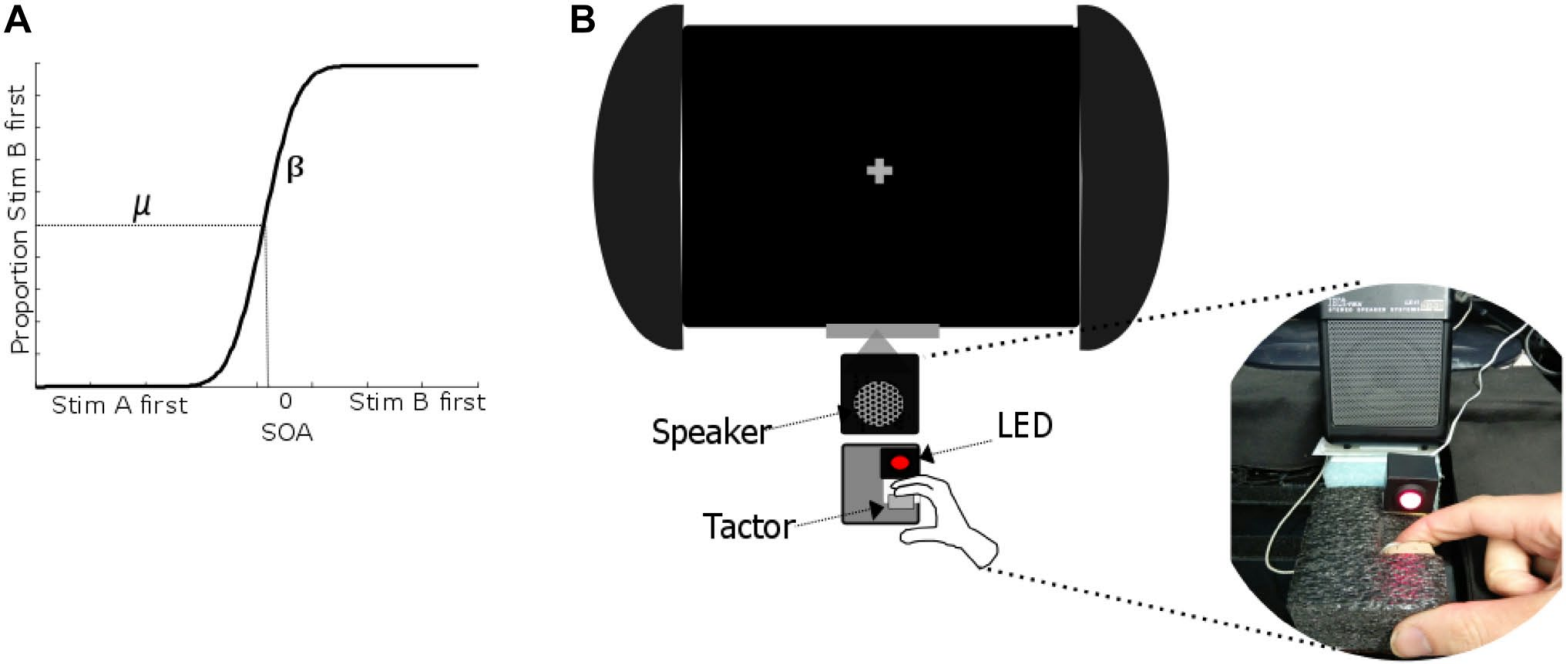
**a** The proportion of trials where the participant judged stimulus B as first is plotted for each SOA and the data is fitted to a Psychometric function. Two parameters were left free to vary in the fitting process: µ gives the mean of the fitted curve (0.5 point, referred to as the Point of Subjective Simultaneity; PSS), and β gives the slope, which is a measure of sensitivity and can be used to calculate the Just Noticeable Difference (JND). **b** Schematic of the experimental set-up for a right handed participant. The participant held a tactor, which was embedded in a foam cube using the thumb and forefinger of their dominant hand. An LED was positioned next to the tactor on the foam cube. The participant was instructed to fixate on a grey cross positioned 19 cm above the speaker

Simultaneity judgement and Temporal Order Judgement (TOJ) tasks are commonly used to measure temporal acuity across sensory modalities (Stone et al. 2001; Vroomen et al. 2004). In a simultaneity judgement task participants are presented with crossmodal stimuli separated by a range of stimulus onset asynchronies (SOAs) and judge whether the stimuli were simultaneous or sequential. Alternatively, in a typical crossmodal TOJ task the participant is presented with stimuli from two different sensory modalities separated by a range of SOAs and asked to judge which stimulus came first. At shorter SOAs, it is more difficult to determine the order of presentation. The participant’s data can be fitted by a Psychometric function to extract measures of temporal acuity between the senses (typically the Just Noticeable Difference; JND, where a smaller JND represents increased acuity), and bias towards a particular sense (Point of Subjective Simultaneity; PSS, which indicates the separation between the stimuli at which the person perceives them to be simultaneous. See Fig. 1a). Previous studies of crossmodal TOJs in neurotypical participants (NTs) have indicated that the PSS varies between individuals and can be influenced by attention to a particular modality (Spence et al. 2001; Stone et al. 2001). That is, if a participant is attending to the modality of stimulus A then it can be presented later than stimulus B, but perceived as simultaneous because the participant will process stimulus A more quickly.

Temporal acuity to crossmodal stimuli appears to be affected over the lifetime in NTs. Visual-auditory simultaneity judgement tasks, have revealed that the temporal acuity of children and adolescents is reduced in comparison to young adults (Hillock et al. 2011; Hillock-Dunn and Wallace 2012). It therefore seems plausible that differences in the developmental trajectory of ASC could affect the maturation of this process. Indeed, reduced temporal acuity for visual-auditory stimuli has been observed across a range of task types (see Stevenson et al. 2015). For instance, children and adolescents with ASC have previously completed simultaneity judgement tasks (Stevenson et al. 2014), and TOJ tasks (de Boer-Schellekens et al. 2013) with stimuli of varying complexity. Participants made judgements regarding simple flash-beeps, speech stimuli and complex, but non-social visual-auditory stimuli. Both studies indicated that acuity was reduced with increasing stimulus complexity. Participants with ASC had reduced acuity (larger JNDs) compared to controls (although this effect was only observed for speech stimuli in the Stevenson et al. 2014 study). Reduced temporal acuity to simple stimuli is associated with poorer visual-auditory speech perception in ASC (Stevenson et al. 2014), which may suggest that low-level differences in temporal processing of crossmodal stimuli can impact on higher level communication issues in ASC.

The temporal alignment between the senses can also be inferred from selective attention tasks in which participants make judgements about a stimulus in a particular modality, while ignoring distracting information presented in a second modality. The extent of temporal separation between the stimuli typically determines whether the stimuli will interact; stimuli which occur closer in time to the target being more likely to influence the participant’s response (Shams et al. 2002; Shore et al. 2006). Studies which have investigated the effects of auditory information on visual judgements across different delays have revealed differences in how NTs and individuals with ASC are affected by temporal separation. Children with ASC were influenced by crossmodal distractors over a range of SOAs twice the size of that for NTs (Foss-Feig et al. 2010; Kwakye et al. 2011).

In the present study, we sought to extend the characterisation of the temporal aspects of multisensory processing in ASC and explore the extent to which multisensory processing may be related to sensory reactivity. The majority of previous research has focused on visual-auditory processing in ASC, but sensory differences associated with touch and hearing are frequently reported (Kern et al. 2006). Consequently, it is important to explore whether any deficits in temporal processing in ASC are specific to visual-auditory interactions, or can also be observed for other bimodal pairings. Adult participants completed TOJs for low level visual-auditory, tactile-auditory and auditory-tactile pairings. TOJ tasks rather than simultaneity judgement tasks were used since apparently reduced temporal acuity in a simultaneity judgement task could be produced by a stronger inclination to report stimuli presented in close temporal proximity as simultaneous (response bias). It was anticipated that temporal acuity would be reduced in participants with ASC in comparison to controls (increased JNDs for each bimodal pairing). There was no directional hypothesis for differences in PSSs between the groups, since differences have not been reported in the literature. Finally, we investigated whether experimental measures (JNDs and PSS) of multisensory temporal processing were related to self-report of sensory reactivity across the groups. We anticipated that increased JNDs would predict more atypical sensory reactivity as this would indicate a reduced ability to separate crossmodal stimuli in time which could lead to perceptually overwhelming experiences. Similarly increased PSS might predict sensory reactivity as this would indicate a bias (or increased attention) towards a particular sense.

## Methods

### Participants

ASC (n = 18) and NT control (n = 18) participants were matched for age, IQ, gender and handedness (see Table 1 for demographic information). Four participants with ASC and four controls were female. One participant with ASC and one NT control were left-handed as self-reported using the Edinburgh Handedness Inventory (Oldfield 1971). All participants had a full scale IQ > 80 as measured using the Wechsler Abbreviated Scale of Intelligence (Wecshler 1999). The diagnosis of ASC participants was confirmed using module 4 of the Autism Diagnostic Observation Schedule (ADOS-2; Lord et al. 2000) by a certified assessor. All participants had normal or corrected to normal vision (6/6 vision in both eyes as measured using Snellens test of visual acuity). To assess sensory reactivity all participants completed the Glasgow Sensory Quotient (GSQ; Robertson and Simmons 2013). The GSQ correlates strongly with autistic traits in both ASC and NT individuals and as such has been recommended as the most suitable instrument for measuring sensory reactivity in ASC (Horder et al. 2014).

**Table 1:** Participant characteristics

		ASC (n = 18)	NT (n = 18)	t (34)	*p*
	Age	31 ± 8.43	31.05 ± 8.71	0.02	.985
	FSIQ	116.56 ± 9.67	112.18 ± 7.56	1.49	.147
	ADOS	8.55 ± 2.28	–	–	–
GSQ score (Bonferonni corrected, α = .013)	Total	76.06 ± 24.28	31.44 ± 17.01	6.38	<.001
	Hyper sensitivity	38.56 ± 14.47	16.78 ± 9.65	5.31	<.001
	Hypo sensitivity	37.50 ± 12.42	14.67 ± 8.83	6.38	<.001

### Apparatus and Stimuli

Participants sat at a desk in a dimly lit room and were instructed to focus on a central fixation point consisting of a grey cross (10 mm) in the centre of the screen. All stimuli were controlled through a PC running E-Prime (Psychology Software Tools Inc., USA). A speaker was used to present sound files (sine wave, 440 Hz, 0.8 AMPs) through a Tacamp amplifier (Dancer Design, St. Helens UK). Auditory stimuli comprised a single 8 ms beep. The speaker was placed on a foam cube to prevent the participants feeling vibrations emitted by the speaker through the table. A 65 × 85 mm foam block was positioned 25 mm in front of the speaker. A bone conductor (Oticon Limited, B/C 2-PIN, 100 Ω, Hamilton, UK) which was driven by the same sound files was embedded in the foam cube and attached to the participant’s index finger on their dominant hand using double sided adhesive. Tactile stimuli comprised a single vibration of 8 ms. A single red LED which subtended a visual angle of approximately 2.24∘ was embedded in a black plastic cube (25 mm) positioned at the tip of the participant’s index finger. Visual stimuli comprised a single 8 ms flash (see Fig. 1b for apparatus).

White noise (~75dB SPL) was played through headphones throughout the experiment to prevent the participant from hearing sounds emitted by the bone conductor. All stimuli were clearly supra-threshold as confirmed by each participant before beginning the experiment.

### Procedure

Participants were asked to report which stimulus came first for each bimodal pairing and their verbal response was recorded by the experimenter.

Stimuli were presented at ±28, 63, 98, 208 and 408 ms SOAs (Poliakoff et al. 2006; Spence et al. 2003; Zampini et al. 2003a, b, 2005). Each trial began with the onset of the central fixation cross followed by a delay randomly selected from a uniform distribution of 500–1000 ms (to prevent additional temporal cues). Then participants were randomly presented with either a visual-auditory, tactile-visual or auditory-tactile pairing. Each of the 30 possible trial types was presented twice in each block. There were five blocks, meaning that each SOA was presented 10 times and there were 300 trials in total. There was a delay of 1000 ms after the experimenter entered the participant’s response, during which time the screen went blank, before the central fixation cross appeared to indicate the next trial was due to commence. Before beginning the main experiment, participants completed 4 trials for each bimodal pairing with the SOA at ±408 ms as practice. These trials included feedback and the participant continued to the main experiment once performance for each pairing was at 75 % accuracy, confirming that all stimuli were suprathreshold and the task instructions understood.

### Data Analysis

Responses for each stimulus pairing were converted to proportion of vision first (visual-auditory trials), tactile first (tactile-visual trials) and auditory first (auditory-tactile trials). Each participant’s data was then fitted by a cumulative Gaussian Psychometric function using the Palamedes toolbox for MATLAB (Kingdom and Prins 2009). Each participant’s JND ($\frac{0.675}{\beta }$; Zampini et al. 2003a, b, 2005) and PSS were then extracted for each stimulus pairing (See Fig. 1b). Individual JND and PSS values were removed prior to analysis where the standard deviation ($\frac{1}{\beta }$) of the psychometric function was larger than the range of SOAs presented (visual-auditory ASC n = 1, auditory-tactile ASC n = 1; see Spence et al. 2001 in which similar exclusion criteria were used). A measure of the goodness of fit of each function was estimated (pDev, range from 0 to 1 with values closer to 1 representing better fits; see Table 2 for group averages). Further data points were removed from analysis where pDev < 0.05 (Kingdom and Prins 2009). It is unlikely that poorly fitted functions, or those with an SOA beyond the stimulus range, gave a reasonable estimate of the participant’s underlying sensory mechanisms.

**Table 2:** Measures of psychometric function goodness of fit (mean pDev ± SD) for each modality pairing where values closer to 1 represent better fits. The final sample size in each condition is also included

	pDev	Final n
Visual-auditory		
ASC	0.29 ± 0.165	15
NT	0.34 ± 0.23	15
Tactile-visual		
ASC	0.45 ± 0.29	18
NT	0.46 ± 0.30	16
Auditory-tactile		
ASC	0.41 ± 0.31	14
NT	0.37 ± 0.21	12

As the remaining samples contained a number of missing cases a Kruskall-Wallis test was then conducted to compare the JND and PSS in each condition between the groups.

Bayes factors were also calculated to compare the strength of the evidence for our principle research hypothesis (participants with ASC would produce higher JNDs than NT participants in each condition) relative to that for the null hypothesis (no difference in JNDs between the groups). Bayesian independent samples t-tests with default priors were conducted comparing JNDs between the groups in each condition using JASP (https://jasp-stats.org/). A Bayes factor measuring evidence for the research hypothesis over the null hypothesis given the observed data is usually denoted BF_10_. The value BF_10_ indicates how many times more likely the research hypothesis is than the null hypothesis. Accordingly a value of BF_10_ = 1 suggests the evidence does not favour either the research or the null hypothesis. Increasingly large values of BF_10_ > 1 suggest greater evidence for the research evidence over the null, whereas decreasing values of BF_10_ < 1 suggest increasing evidence for the null hypothesis over the research hypothesis (Dienes 2014).

As an exploratory analysis, multiple regressions were calculated for each group to explore the relationship between sensory scores reported using the GSQ and participants JND and PSS in each condition.

## Results

**Fig. 2:**
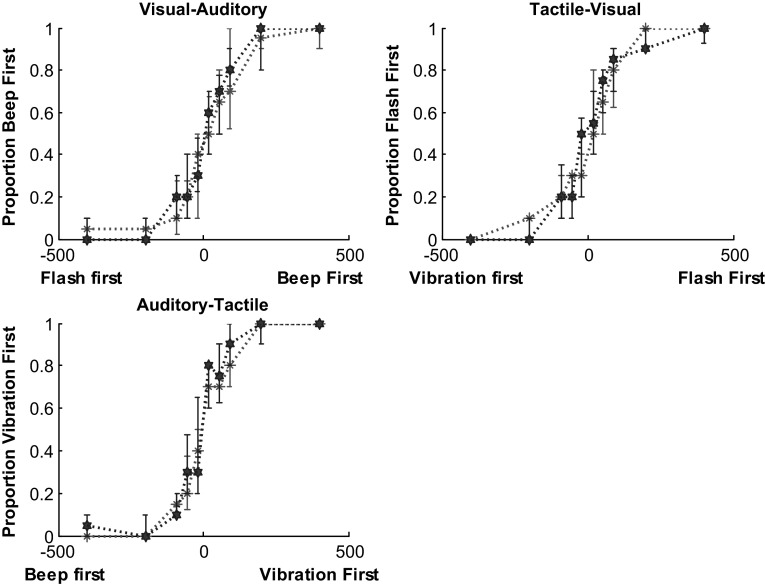
Median responses at each SOA for each bimodal pairing. The data points for the ASC group are represented by *red asterisks* and the NT group are represented by the *blue stars. Error bars* denote the interquartile range. Note that individual fitted functions were used to calculate the JNDs and PSSs used in the analysis as functions fitted at the group level lack the sensitivity to detect individual differences in performance

Each group’s median responses to stimuli presented at each SOA for each modality pairing are given in Fig. 2.

### Just Noticable Difference

**Fig. 3:**
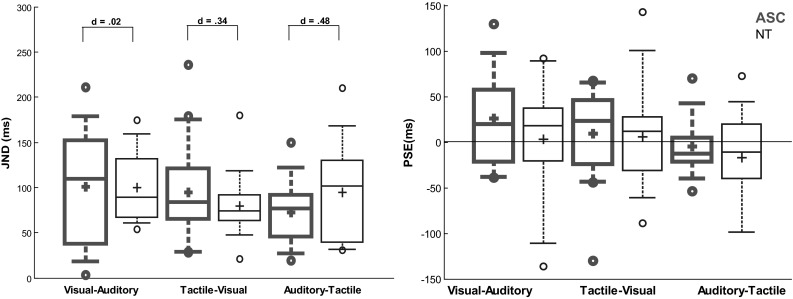
JND and PSSs for each bimodal pair. Participants with ASC are represented in *bold red* and NT in *blue*. The edges of each *box* represent the upper and lower quartile and the *whiskers* represent the most extreme deciles. The *line* within the *box* represents the median in each condition and the cross is the mean. The *open dots* represent the most extreme data points. Cohens d is given for comparison between the JNDs for participants with ASC and NT in each condition. For PSS values, a negative value indicated that the first listed modality in that pairing was presented first e.g. vision was presented first for visual-auditory values

The size of the JNDs did not differ between participants with ASC or NT for any modality pairing (see Fig. 3). ASC participant JNDs were larger than NTs for the visual-auditory (χ^2^ (1, n = 30) < 0.01, *p* = .950) and for tactile-visual (χ^2^ (1, n = 34) = 1.22, *p* = .270) modality pairings, but these differences did not reach statistical significance. The JNDs of NT participants were larger than the ASC group for the tactile-auditory modality pairings (χ^2^ (1, n = 26) = 1.17, *p* = .280), but this did not reach statistical significance. A Bayes Factor (BF) was calculated to compare JNDs between the groups for each modality pairing. For visual-auditory JNDs BF_10_ = 0.35 meaning that the research hypothesis was 0.35 times more likely than the null given the data (or equivalently that the null was 2.82 times more likely than the research hypothesis). For tactile-visual JNDs BF_10_ = 0.74 meaning the research hypothesis was 0.74 times more likely than the null given the data (or that the null was 1.34 times more likely). For auditory-tactile JNDs BF_10_ = 0.19 meaning the research hypothesis was 0.19 times more likely than the null given the data (or that the null was 5.28 times more likely).

**Fig. 4:**
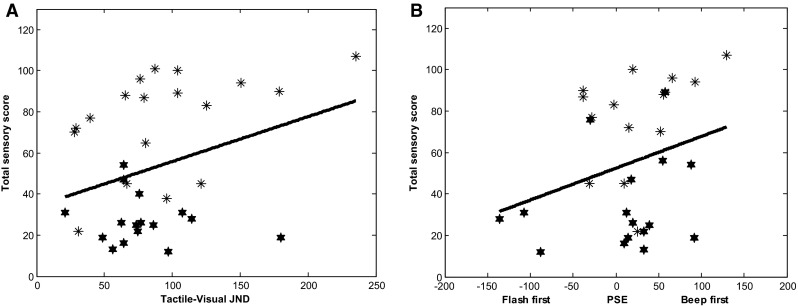
Tactile-Visual JNDs (**a**) and PSS in the visual-auditory condition (**b**) plotted as a function of participants GSQ score. Data points for the ASC group are represented by the *asterisks* and the NT group by *stars*

A multiple regression was calculated exploring the relationship between self-reported sensory reactivity score as measured using the GSQ and the JND for each bimodal pairing (Table 3). The regression model was not statistically significant (F (3, 19) = 2.20, *p* = .128). However, JNDs for the visual-tactile modality pairing were a significant predictor of sensory reactivity, indicating that participants who had reduced temporal acuity to tactile-visual stimuli also reported more atypical sensory reactivity. A follow-up Pearson’s correlation coefficient revealed a non-significant positive correlation between tactile-visual JNDs and sensory reactivity scores (r (34) = .323, *p* = .063). See Fig. 4a.

**Table 3:** Regression data comparing JND and PSS in each modality pairing with total sensory scores

	R^2^ adjusted	β	t	p
JND	.16			
Visual-auditory JND		.24	0.59	.562
Tactile-visual JND		.65	2.14	.048*
Auditory-tactile JND		.04	0.12	.903
PSS	.16			
Visual-auditory PSS		.68	2.56	.021*
Tactile-visual PSS		.17	0.74	.473
Auditory-tactile PSS		.35	1.39	.184

Significant predictors are highlighted with an asterisk

### Point of Subjective Simultaneity

There were no statistically significant differences in the PSS between the groups for any of the modality pairings (see Fig. 3). The ASC participants exhibited a more positive PSS for the visual-auditory pairings than NTs, but this difference was not statistically significant (χ^2^ (1, n = 30) = 0.36, *p* = .548). For the tactile-visual pairing, the ASC participants were more positive than the NTs, but this difference was not statistically significant (χ^2^ (1, n = 34) = 0.57, *p* = .448). For the auditory-tactile pairing, NT participants were more negative, but this difference was not statistically significant (χ^2^ (1, n = 26) = 0.04, *p* = .837).

A multiple regression was conducted to explore the relationship between self-reported sensory reactivity and the PSS for each bimodal pairing (Table 3). The regression model revealed that PSS scores overall did not significantly predict sensory reactivity (F (3, 19) = 2.21, *p* = .127). However, PSS scores in the visual-auditory condition were a significant positive predictor of sensory reactivity, indicating that participants who required the auditory stimulus to be presented prior to the visual stimulus in order to judge the stimuli as simultaneous reported more atypical sensory reactivity (see Fig. 4). A follow-up Pearson’s correlation coefficient revealed a non-significant positive correlation between visual-auditory PSS and sensory reactivity scores (r (30) = .29, *p* = .129).

## Discussion

The aim of the current study was to explore temporal acuity between the senses in adults with ASC. The data suggest that temporal acuity (JND) does not differ between the groups for any modality pairing, although there was greater variability in performance in the ASC group. The data also suggest that there are no between group differences in bias towards a stimulus (PSS). Interestingly, both the JND in the visual-tactile condition and the PSS in the visual-auditory condition predicted self-reported sensory reactivity.

The findings of the current study contrast with previous research suggesting that temporal acuity is reduced for simple visual-auditory stimuli in children and young adults with ASC (de Boer-Schellekens et al. 2013; Stevenson et al. 2014). As visual-auditory temporal acuity is believed to be reduced in ASC, it is interesting that in the present study the effect size was smallest (d = 0.02) for the between group comparison of visual-auditory JNDs. This suggests that temporal acuity is comparable to NTs in some adults with ASC (see de Boer-Schellekens et al. 2013; Poole et al. 2015 for similar recent findings). As multisensory temporal acuity typically matures across development (Hillock et al. 2011; Hillock-Dunn and Wallace 2012) there may be a delay in the maturation of this processing in ASC which may have ‘caught up’ with NT performance by adulthood (see Foxe et al. 2015; Taylor et al. 2010, for evidence of maturation of visual-auditory speech processing in adolescents with ASC). However, the variability in the ASC group’s performance is worth noting. For example, the most extreme values for visual-auditory JNDs (Fig. 3) display a participant close to 0 and another over 200 ms. In addition, a participant with ASC excluded from analysis produced a standard deviation beyond the range of SOAs which could suggest very poor acuity for visual-auditory stimuli (although see below for a possible limitation in the selection of SOA). This variability in acuity may reflect individual differences in the development of multisensory processing, or in the use of compensatory strategies.

The present study is the first to explore differences in multisensory temporal acuity in ASC across multiple modality pairings, but there are some limitations. The SOAs used were based on previous TOJ studies in NT participants. However, differences between ASC and NT performance on visual tasks including task-irrelevant auditory information have previously revealed between group differences for stimuli presented between 150 ms-300 ms SOA (Foss-Feig et al. 2010; Kwakye et al. 2011). As only a single SOA was presented within this range, it is possible that the current experiment was not optimised to draw out differences between the groups. Similarly, there were only 10 repetitions of each SOA which may have contributed to a number of participant’s functions being poorly fitted. Nevertheless, the current investigation suggests that temporal acuity is not universally impaired in adults with ASC. There is a need for further studies exploring temporal acuity between the senses across adolescence and into early adulthood in both NTs and in ASC.

The current findings revealed novel relationships between aspects of multisensory temporal processing and self-reports of sensory reactivity. In the tactile-visual condition participant JNDs were a significant predictor of self-reported sensory reactivity, such that those with reduced tactile-visual temporal acuity reported greater sensory reactivity. Reduced tactile-visual temporal acuity could lead to atypical experiences of touch which could impact on higher level processes such as the experience, and use, of inter-personal touch (Poole et al. 2015) and in planning movements, particularly involving objects (Gowen and Hamilton 2013). Furthermore, participants who required a greater auditory lead to perceive visual-auditory stimuli as simultaneous reported more atypical sensory reactivity. Counterintuitively, the auditory lead suggests a bias *towards* visual information as it means that the auditory stimulus must be presented *before* the visual stimulus in order for them to be judged as simultaneous; i.e. the visual stimulus is processed more quickly. Previous research in NTs has suggested that the PSS can be biased towards an attended stimulus and is processed more quickly (Spence et al. 2001; Spence and Parise 2010) and selective attention has been implicated in TOJ tasks (Binder, 2015). The current finding therefore suggests that increased attention towards visual information may also contribute to sensory reactivity. Indeed, recent studies have observed differences in the selective attention to vision in ASC (Murphy et al. 2014; Occelli et al. 2013). The finding that a visual bias predicted sensory traits is partially consistent with findings from a visual-auditory simultaneity judgement task in NTs, which indicated that a shift in PSS towards the auditory stimulus was correlated positively with autistic traits (Donohue et al. 2012 1). That is, those with higher autistic traits showed a similar pattern to the participants in the current study with more atypical sensory reactivity.

As previous investigations have indicated that the temporal acuity of crossmodal stimuli can be enhanced with experience (Donohue et al. 2010), and training (Powers et al. 2009; Stevenson et al. 2013; Vroomen et al. 2004) the current findings suggest that such approaches may be effective in reducing sensory reactivity in ASC.

In summary, the current investigation provided no evidence for reduced temporal acuity to crossmodal stimuli in adults with ASC. It may be that there is a developmental delay in the maturation of this process which has ‘caught up’ with NT performance by adulthood. However, the variability in the ASC data suggests that there are important individual differences in temporal processing of crossmodal stimuli, which merit further investigation. Indeed, the tactile-visual JND and visual-auditory PSS data were predictors of sensory reactivity. Participants with reduced tactile-visual temporal acuity and those who were more biased towards vision for visual-auditory judgements reported more sensory reactivity. This preliminary finding suggests that both reduced tactile-visual temporal acuity and atypical selective attention to vision warrant further investigation to understand and ameliorate atypical sensory reactivity.

## Footnotes

1 Although the authors of this study erroneously interpreted this effect as a bias towards auditory information.

## References

[CR1] de Boer-Schellekens L., Eussen M., Vroomen J. (2013). Diminished sensitivity of audiovisual temporal order in autism spectrum disorder. Frontiers in Integrative Neuroscience.

[CR2] de Boer-Schellekens L., Keetels M., Eussen M., Vroomen J. (2013). No evidence for impaired multisensory integration of low-level audiovisual stimuli in adolescents and young adults with autism spectrum disorders. Neuropsychologia.

[CR3] Dienes Z. (2014). Using Bayes to get the most out of non-significant results. Frontiers in Psychology.

[CR4] Donohue S. E., Darling E. F., Mitroff S. R. (2012). Links between multisensory processing and autism. Experimental Brain Research.

[CR5] Donohue S. E., Woldorff M. G., Mitroff S. R. (2010). Video game players show more precise multisensory temporal processing abilities. Attention, Perception & Psychophysics.

[CR6] Foss-Feig J. H., Kwakye L. D., Cascio C. J., Burnette C. P., Kadivar H., Stone W. L., Wallace M. T. (2010). An extended multisensory temporal binding window in autism spectrum disorders. Experimental Brain Research.

[CR7] Foxe J. J., Molholm S., Del Bene V. A., Frey H. P., Russo N. N., Blanco D., Saint-Amour D., Ross L. A. (2015). Severe multisensory speech integration deficits in high-functioning school-aged children with autism spectrum disorder (ASD) and their resolution during early adolescence. Cerebral Cortex.

[CR8] Gowen E., Hamilton A. (2013). Motor abilities in autism: A review using a computational context. Journal of Autism and Developmental Disorders.

[CR9] Hillock A. R., Powers A. R., Wallace M. T. (2011). Binding of sights and sounds: Age-related changes in multisensory temporal processing. Neuropsychologia.

[CR10] Hillock-Dunn A., Wallace M. T. (2012). Developmental changes in the multisensory temporal binding window persist into adolescence. Developmental Science.

[CR11] Horder J., Wilson C. E., Mendez M. A., Murphy D. G. (2014). Autistic traits and abnormal sensory experiences in adults. Journal of Autism and Developmental Disorders.

[CR12] Iarocci G., McDonald J. (2006). Sensory integration and the perceptual experience of persons with autism. Journal of Autism and Developmental Disorders.

[CR13] Kern J. K., Trivedi M. H., Garver C. R., Grannemann B. D., Andrews A., Savla J. S., Johnson D. G., Mehta J. G., Schroeder J. L. (2006). The pattern of sensory processing abnormalities in autism. Autism: The International Journal of Research and Practice.

[CR14] Kingdom F. A. A., Prins N. (2009). Psychophysics: A practical introduction.

[CR15] Kwakye L. D., Foss-Feig J. H., Cascio C. J., Stone W. L., Wallace M. T. (2011). Altered auditory and multisensory temporal processing in autism spectrum disorders. Frontiers in Integrative Neuroscience.

[CR16] Lane A. E., Young R. L., Baker A. E. Z., Angley M. T. (2010). Sensory processing subtypes in autism: Association with adaptive behavior. Journal of Autism and Developmental Disorders.

[CR41] Lord, C., Risi, S., Lambrecht, L., Cook, E. H., Leventhal, B. L., DiLavore, P. C., Pickles, A. & Rutter, M. (2000). The autism diagnostic observation schedule-generic: A standard measure of social and communication deficits associated with the spectrum of autism. *Journal of Autism and Developmental Disorders, 30*(3), 205–223. 11055457

[CR17] Murphy J. W., Foxe J. J., Peters J. B., Molholm S. (2014). Susceptibility to distraction in autism spectrum disorder: Probing the integrity of oscillatory alpha-band suppression mechanisms. Autism Research.

[CR18] O’Neill, M., & Jones, R. S. (1997). Sensory-perceptual abnormalities in autism: A case for more research? *Journal of Autism and Developmental Disorders, 27*(3), 283–293. Retrieved from http://www.ncbi.nlm.nih.gov/pubmed/9229259. 10.1023/a:1025850431170 9229259

[CR19] Occelli V., Esposito G., Venuti P., Arduino G. M., Zampini M. (2013). Attentional shifts between audition and vision in autism spectrum disorders. Research in Autism Spectrum Disorders.

[CR20] Oldfield R. C. (1971). The assessment and analysis of handedness: The Edinburgh inventory. Neuropsychologia.

[CR21] Poliakoff E., Shore D. I., Lowe C., Spence C. (2006). Visuotactile temporal order judgments in ageing. Neuroscience Letters.

[CR22] Poole D., Gowen E., Warren P. A., Poliakoff E. (2015). Investigating visual–tactile interactions over time and space in adults with autism. Journal of Autism and Developmental Disorders.

[CR23] Powers A. R., Hillock A. R., Wallace M. T. (2009). Perceptual training narrows the temporal window of multisensory binding. The Journal of Neuroscience.

[CR42] Robertson, A. E., & Simmons, D. R. (2013). The relationship between sensory sensitivity and autistic traits in the general population. *Journal of Autism and Developmental Disorders, 43*(4), 775–784. doi:10.1007/s10803-012-1608-7. 10.1007/s10803-012-1608-7 22832890

[CR24] Shams L., Kamitani Y., Shimojo S. (2002). Visual illusion induced by sound. Cognitive Brain Research.

[CR25] Shore D. I., Barnes M. E., Spence C. (2006). Temporal aspects of the visuotactile congruency effect. Neuroscience Letters.

[CR26] Spence C., Baddeley R., Zampini M., James R., Shore D. I. (2003). Multisensory temporal order judgments: When two locations are better than one. Perception & Psychophysics.

[CR27] Spence C., Parise C. (2010). Prior-entry: A review. Consciousness and Cognition.

[CR28] Spence C., Shore D., Klein R. (2001). Multisensory prior entry. Journal of Experimental Psychology.

[CR29] Stevenson, R. A., Segers, M., Ferber, S., Barense, M. D., Camarata, S., & Wallace, M. T. (2015). Keeping time in the brain: Autism spectrum disorder and audiovisual temporal processing. Autism Research, 24(9). doi:10.1002/aur.1566. 10.1002/aur.1566 26402725

[CR30] Stevenson R. A., Siemann J. K., Schneider B. C., Eberly H. E., Woynaroski T. G., Camarata S. M., Wallace M. T. (2014). Multisensory temporal integration in autism spectrum disorders. The Journal of Neuroscience.

[CR31] Stevenson R. A., Siemann J. K., Woynaroski T. G., Schneider B. C., Eberly H. E., Camarata S. M., Wallace M. T. (2014). Evidence for Diminished Multisensory Integration in Autism Spectrum Disorders. Journal of Autism and Developmental Disorders.

[CR32] Stevenson R. A., Wilson M. M., Powers A. R., Wallace M. T. (2013). The effects of visual training on multisensory temporal processing. Experimental Brain Research.

[CR33] Stone J. V., Hunkin N. M., Porrill J., Wood R., Keeler V., Beanland M., Portn M., Porter N. R. (2001). When is now? Perception of simultaneity. Proceedings of the Royal Society.

[CR34] Taylor N., Isaac C., Milne E. (2010). A comparison of the development of audiovisual integration in children with autism spectrum disorders and typically developing children. Journal of Autism and Developmental Disorders.

[CR35] Vroomen J., Keetels M., de Gelder B., Bertelson P. (2004). Recalibration of temporal order perception by exposure to audio-visual asynchrony. Brain Research.

[CR36] Wechsler D. (1999). Wechsler abbreviated scale of intelligence.

[CR37] Zampini M., Brown T., Shore D. I., Maravita A., Röder B., Spence C. (2005). Audiotactile temporal order judgments. Acta Psychologica.

[CR39] Zampini, M., Shore, D. I., & Spence, C. (2003a). Audiovisual temporal order judgments. *Experimental Brain Research, 152*(2), 198–210. doi:10.1007/s00221-003-1536-z. 10.1007/s00221-003-1536-z 12879178

[CR40] Zampini, M., Shore, D. I., & Spence, C. (2003b). Multisensory temporal order judgments: The role of hemispheric redundancy. *International Journal of Psychophysiology, 50*(1–2), 165–180. doi:10.1016/S0167-8760(03)00132-6. 10.1016/s0167-8760(03)00132-6 14511844

